# Two Cases of Hæmophilia

**Published:** 1884-12

**Authors:** 


### TWO CASES OF HÆMOPHILIA. By the Editor.

These two cases, which have been under my observation during the past year, present, in conjunction, a very complete clinical study of the Hæmorrhagic Diathesis, or Hæmophilia.

*Case* 1.—The first patient, an exceedingly delicate lad of 17, was admitted to the Bristol Infirmary in February,

1884, with bleeding from a scratch on the ball of the thumb, which had been going on for three weeks. All the orthodox plans of treatment appeared to have been tried, and the result was a sloughy sore, as big as a florin, from which the blood welled up freely on the removal of pressure. I stopped all pressure, put him to bed, elevated the arm, and applied a simple dressing of compound tincture of benzoin. The bleeding diminished considerably, but still continued after three days. I then applied Ruspini's styptic locally, and in two days it had quite stopped, and the wound rapidly granulated up. Large doses of the perchloride of iron and of gallic acid were given internally.

After a fortnight he appeared to be in his usual state of health, when, after a restless night, he awoke with his right elbow-joint very painful and full of fluid. In three days this fluid disappeared, and the joint recovered its ordinary mobility.

The blood which came from the wound was of the ordinary watery nature after prolonged hæmorrhage. Tested by Gomers's Hæmactyometer, the hæmoglobin was found to be $\frac{6}{10}$ of the normal, and the corpuscles 1,600,000, instead of 5,000,000.

This boy had had a large and varied experience of hospitals and surgeons. When fifteen months old he was in St. Thomas's Hospital for a swelling of the knee, which appears to have been called white swelling. When eight years old he was in the Bristol Children's Hospital with a swelling in the same joint, to which the same name was given. When eleven he was in the Bristol Infirmary, under the late Mr. Tibbits, with fluid in several of his joints. The condition was then diagnosed as one of hæmophilia, and one of his joints was aspirated, pure blood being removed, but almost immediately refilled. So peculiar was his appearance, with wasted limbs and swollen, distorted joints, that he was photographed. Since then he has been frequently a patient at the Infirmary with bleedings of various sorts, spontaneous from the nose and lips, or traumatic from scratches or cuts.

The most prominent clinical feature of the case is the joint-swelling. Every large joint in his body has been, at one time or another, affected; and on every one of the frequent occasions when I have seen him, at least two of his joints have been distended with a fluid, which is, doubtless, blood. Usually the joint fills spontaneously in a few hours, causing him much pain, and subsides in a few days. Sometimes a twist or wrench is the cause of the hæmarthros. Even rough handling may be a cause. For instance, he was exhibited at a local medical meeting, and his wrist was several times grasped to move his elbow, which was full of fluid. Next day his wrist-joint also was full of fluid, and the tissues around were ecchymosed. With him a sharp tap will cause an ecchymosis.

Occasionally he appears at the Infirmary with a "taste of blood in his mouth," from oozing through the lips or gums. Blood has not been observed in urine or fæces, and it is now some time since he has had bleeding from the nose.

The heredity of the complaint is, as usual, on the mother's side, though, in spite of careful inquiries, no more than one generation can be accounted for. One brother died in childhood, of bleeding from the opening by incision of a small abscess of the scalp. Four sisters and three brothers have grown up strong and well without showing any signs of the diathesis. His mother had a brother who died of bleeding. These are all the facts bearing on history which the meagre knowledge of the family about their relatives can supply.

*Case 2.*—This was a child fifteen months old, who was admitted in July, 1884, for bleeding from superficial abrasions on his finger and palm, caused by a fall. This happened twelve days before admission. He had had medical advice, and all the ordinary remedies were tried. He was absolutely blanched, looking more like a wax model than a living child, and was very restless, and had begun to vomit. The sores had begun to slough from the pressure exerted.

All pressure was removed, the arm was fixed on a splint, and simple turpentine dressing applied. The bleeding at once stopped, and the wounds soon healed up. At the end of three weeks he was discharged, having gained strength with rapidity.

This was the first attack of bleeding which the child had had; the mother believed it was the first wound he had received. The child had not shown signs of constitutional weakness, but had always been liable to get extensive bruises from trivial causes. In fact, he was never without one or more areas of ecchymosis. There had been no joint-swelling or bleeding from any of the mucous membranes.

The diathesis here also is on the mother's side. Two maternal uncles died of hæmorrhage; one in childhood, from biting his tongue; the other at twenty-four, after the extraction of a tooth. Farther back than this the family records cannot be traced. He has one brother, aged twelve, who is a bleeder and also suffers from hæmorrhages under the skin, and from the mucous membranes of the nose and mouth. No other relatives have shown signs of bleeding.

I have seen three other cases of hæmorrhagic diathesis. One was in consultation with Dr. Lionel A. Weatherly, of Portishead, where there was severe episataxis. This patient had had blood in two at least of his joints, and bled from the kidneys as well. Bleeding from ordinary cuts and scratches were usually stopped without much difficulty. The two others were under the care of colleagues at the Infirmary, and presented no new features.

*Remarks.*—It is needless that I, who have nothing new to say, should add to the already voluminous literature of the subject. Perhaps Dr. Wickham Legg, who has made the subject peculiarly his own, may, in the forthcoming second edition of his work on *Hæmophilia*, help to throw some light on its pathology. So far as the modes of investigation as yet applied can help us, its pathogeny would seem to be neither in the structure of the blood-vessels nor in the composition of the blood. Would it be worth while to examine the nervous system as well? Might it be that the nervous force which causes the arterial muscles to contract is deficient or wanting? Is it a paralysis of the arterial muscles?

As to treatment, I believe that the best plan, if we had the courage to adopt it, would be to throw physic to the dogs, and let the patient alone. Absolutely perfect rest in bed, mentally and bodily, locally and generally, with milk diet, and the simplest of dressings lightly applied, I believe to be all that is required. The actual cautery (*pace* Holmes) is to be unhesitatingly condemned. It makes matters tenfold worse, by substituting a slough with bleeding from big and deep vessels for an abrasion with mere oozing from superficial vessels. Strong pressure, powerful astringents, and everything which tends to destroy weakened tissues, are to be avoided. They tide the patient over the first danger to leave him in a second and greater one. If we must plug the nares to save the patient's life, let the pressure of the plug be of the lightest, and let it be removed at least every twenty-four hours. As a hospital resident, I was actively concerned in the death of a patient through repeated plugging of the nares, and consequent ulceration of the mucous membrane. In a case of true hæmophilia, nothing less than imminent death would make me plug the nares. Lint soaked in turpentine I believe to be the best local application.

Constitutionally, I have administered gallic acid in fifteen-grain doses every three hours, turpentine in drachm doses every two hours, purgative salts and Ruspini's styptic—all during the period of bleeding. In the intervals, perchloride of iron has the best reputation. But, with the exception of the iron, I doubt if equally good results might not be got without any of them. Opium, I think, ought to be always administered during the bleeding. It quiets the restlessness, and so provides at least physical rest.

In conclusion, and specially with reference to the manner in which the disease is inherited, through the maternal grandfather—a peculiarity which it shares with colour blindness—I venture to suggest that we should note any peculiarities in the transmission of other dyscrasiæ or malformations. On working up the literature of such well-known subjects as cleft palate and extroversion of the bladder, I find a remarkable deficiency in the accounts of modes of inheritance; and a more superficial investigation in other directions shows a similar lack of information.

